# Longitudinal analysis of viral and host RNA in blood and saliva during controlled human dengue virus 3 infections

**DOI:** 10.1128/jvi.00373-26

**Published:** 2026-05-14

**Authors:** Sharon S. Wu, Jennifer N. Bjerke, Casey E. Middleton, Arturo Barbachano-Guerrero, Joshua R. Dye, Emma R. Worden-Sapper, Amy B. Emerman, Timothy P. Endy, Stephen J. Thomas, Adam T. Waickman, Daniel B. Larremore, Nicholas R. Meyerson, Sara L. Sawyer

**Affiliations:** 1 Department of Molecular, Cellular, and Developmental Biology, University of Colorado Boulder 315558, Boulder, Colorado, USA; 2 BioFrontiers Institute, University of Colorado Boulder, Boulder, Colorado, USA; 3 Darwin Biosciences, Boulder, Colorado, USA; 4 Department of Computer Science, University of Colorado Boulder 1877, Boulder, Colorado, USA; 5 Global Health Institute, State University of New York Upstate Medical University, Syracuse, New York, USA; 6 Department of Microbiology and Immunology, State University of New York Upstate Medical University, Syracuse, New York, USA; 7 Santa Fe Institute 7203, Santa Fe, New Mexico, USA; Wake Forest University School of Medicine, Winston-Salem, North Carolina, USA

**Keywords:** host response, dengue virus

## Abstract

Dengue viruses are mosquito-borne human pathogens whose global incidence in human infections is rising. Surveillance is hampered by the requirement for blood-based diagnostics. Interestingly, in several prior research studies, dengue virus nucleic acids and proteins could be detected in saliva, making saliva-based diagnostics a plausible alternative. However, the temporal relationships between exposure, viremia, salivary accumulation, and symptom onset remain poorly defined. To address this knowledge gap, nine participants were enrolled in clinical trial NCT04298138 to receive an inoculation with an attenuated dengue virus (DENV-3 strain CH53489). Matched blood and saliva specimens were collected over 10 days. Dengue virus genomes could be detected by RT-qPCR in both blood and saliva before the onset of symptoms in most individuals. The earliest time of detection in blood and saliva was 3.4 and 5.0 days post-infection, respectively. Using this sample set, we also measured host transcriptional responses to dengue infection in both biospecimens. Strikingly, far more human genes showed increased transcript abundance exclusively in saliva (14%) than in PBMCs (5%) or both compartments simultaneously (6%), revealing compartment-specific host responses. For 21 of the human transcripts that increased in response to dengue infection, we quantified their daily abundance across the course of DENV-3 infection in all study participants. Together, these findings demonstrate that sensitive dengue virus detection in saliva should be possible and establish that both blood and saliva capture early host responses to infection.

**IMPORTANCE:**

Dengue virus infections are detected using blood samples, either with nucleic acid amplification tests for the dengue virus genome (in the case of an active infection) or serology-based tests for antibodies recognizing dengue viruses (indicating a prior infection). The requirement for blood draws creates a barrier to dengue virus surveillance. Our data demonstrate that saliva can serve as an alternative biospecimen for detecting dengue virus genomes early in infection, with detection lagging blood by only 1.6 days—a modest and likely acceptable tradeoff given saliva’s practical advantages. Unlike blood, saliva is non-invasive, self-collectible, requires no sharps, and is thermostable. We also identified host genes upregulated upon dengue infection, informing on the host response to infection. Our longitudinal study design enabled us to track the kinetics of transcript accumulation both by day post-infection and relative to symptom onset, providing high resolution into the host response to the virus.

**CLINICAL TRIALS:**

This study is registered with ClinicalTrials.gov as NCT04298138.

## INTRODUCTION

Dengue viruses are blood-borne pathogens of public health concern, especially in tropical and subtropical areas of the world where their mosquito vectors circulate (1). Recently, there have been global increases in dengue virus cases (2–6). The World Health Organization reported 2024 to have the highest number of cases ever reported, double that of the previous year: over 14.6 million cases and 12,000 deaths worldwide (7, 8). There are four distinct dengue viruses circulating in humans, called dengue virus “serotypes” 1, 2, 3, and 4 (or DENV-1, DENV-2, DENV-3, and DENV-4) (9). Dengue virus infection has been recognized as particularly challenging with regards to both vaccination and treatment (10). Regarding diagnosis and surveillance, both acute and prior dengue virus infections are detected using blood samples, either with nucleic acid amplification tests for the dengue virus genome (in the case of an active infection) or serology-based tests for antibodies recognizing dengue viruses (indicating a prior infection) (11–14). Saliva is an attractive alternative biomaterial to blood because it avoids the use of sharps, and is readily accessible (15, 16). While other studies have detected dengue virus genomes and proteins in human saliva (15, 17–21), controlled infections that allow for daily sample collection and longitudinal analysis are lacking. Such longitudinal studies would elucidate the relative timing of exposure, viral genomes becoming measurable in blood and in saliva, and symptom onset. However, precise infection kinetics can only be established when the moment of infection is known—a condition rarely met outside of controlled human trials. In the few studies where humans have been experimentally infected with dengue virus and followed over a time course, only blood was collected (22–24).

Controlled human infection models (CHIMs) are study designs in which healthy, consenting volunteers are deliberately inoculated with a well-defined strain of a pathogen under controlled clinical conditions. Because the timing of infection is known precisely, CHIMs allow researchers to capture the early kinetics of viral replication and the ensuing immune response with a resolution that is simply not achievable in natural infection cohorts. Furthermore, because all participants are inoculated with the same well-characterized strain, CHIMs eliminate the pathogen genetic heterogeneity inherent to natural infections, allowing differences in outcome between participants to be attributed to host factors such as immune status or genetics. CHIMs are also increasingly central to vaccine evaluation: if vaccinated participants resist infection, or exhibit reduced viremia and attenuated clinical symptoms relative to unvaccinated controls, this constitutes a meaningful and interpretable signal of vaccine efficacy (25, 26).

Here, we present the results of a CHIM involving dengue virus infection with parallel collection of blood and saliva. Nine participants were enrolled in clinical trial NCT04298138 to receive a single subcutaneous inoculation of the attenuated DENV-3 strain CH53489. Originally developed as a potential vaccine strain, CH53489 has previously been utilized for human challenge studies to monitor the immune response to dengue infection in a controlled environment (22, 27). Clinical and immunological endpoints of this human challenge study have been published separately (22). We report here on the saliva arm of this study and compare signals of infection in blood versus saliva. We accomplished two things with this study. First, we showed that blood is only slightly superior to saliva for the earliest possible detection of dengue virus infection. Viral RNA was detectable in saliva only 1.6 days later than in blood. Thus, future saliva-based tests could make molecular diagnostics more accessible globally. Second, we used our longitudinal study to acquire new knowledge about the host response to infection. We identified human genes in PBMC and saliva samples with transcripts that are increased upon dengue infection.

## RESULTS

### DENV-3 genomes accumulate with similar kinetics in both blood and saliva

**Fig 1:**
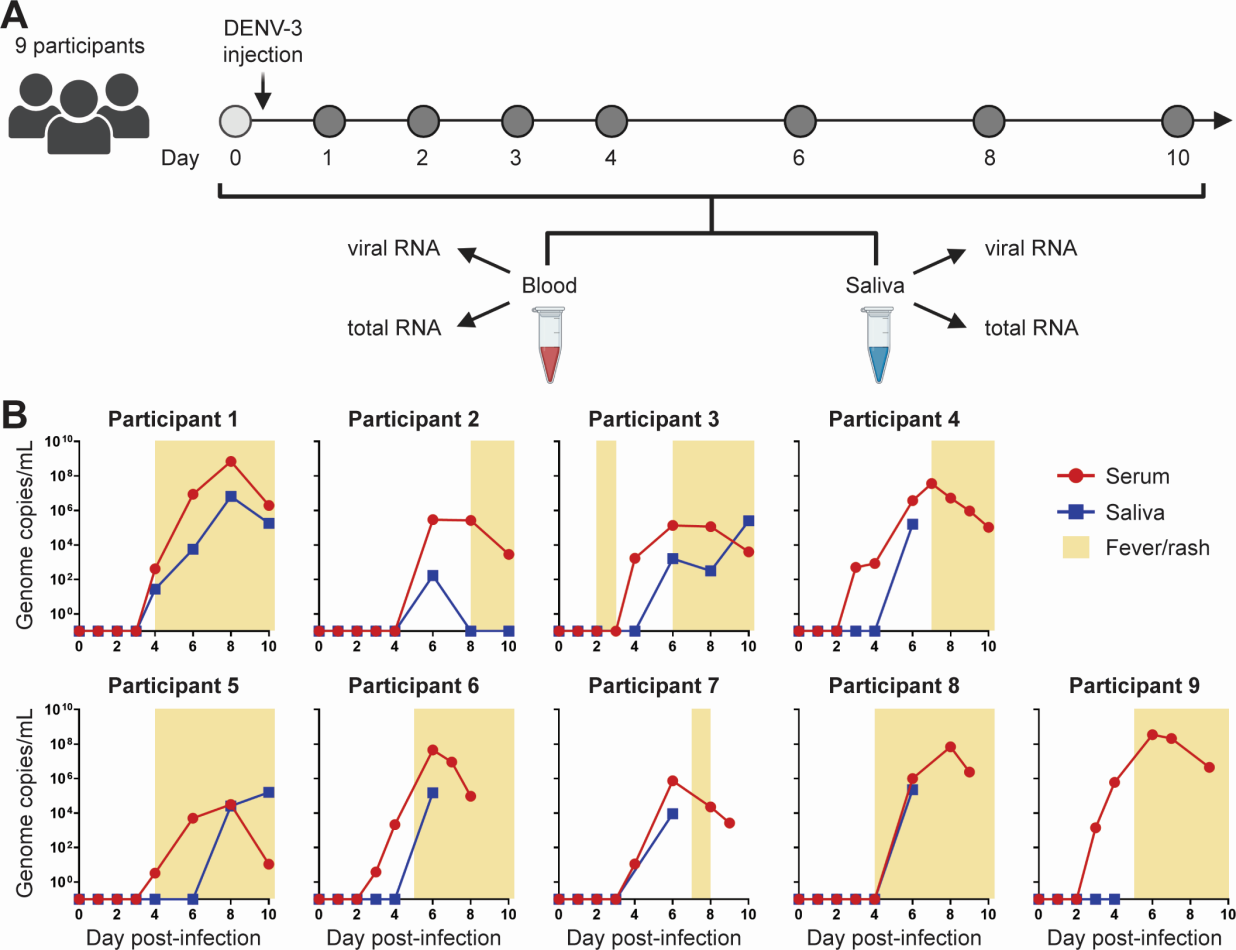
DENV-3 genomes can be detected with similar kinetics and titers in blood and saliva. (**A**) Schematic of the challenge study and samples collected. Nine participants were inoculated with 700 plaque-forming units of DENV-3 strain CH53489. Saliva and blood were collected from participants before DENV-3 infection (day 0) and at days 1, 2, 3, 4, 6, 8, and 10 post-infection. Total RNA or viral RNA was harvested from samples as described in Materials and Methods. (**B**) Viral genomes were quantified by RT-qPCR on RNA from serum (red data; previously published (22) or saliva (blue data). Yellow sections of the graphs denote days each participant experienced fever and/or rash.

Nine participants were enrolled in clinical trial NCT04298138 to receive a single subcutaneous inoculation with 700 plaque-forming units of the attenuated DENV-3 strain CH53489 (27, 28). Enrollees donated blood and saliva in parallel for up to 10 days post-infection in either an outpatient or hospital setting, where symptoms were also clinically assessed (Fig. 1A). A few sampling days were missed by some participants (Table S1). The previous analysis of blood samples from this study demonstrated that all enrollees became infected, seroconverted, and developed memory T-cell responses (22). Here, we performed a quantitative analysis of dengue virus genome accumulation in these two biological fluids. We found that DENV-3 genomes are detectable by RT-qPCR in both serum and saliva, with a delay in saliva relative to serum (Fig. 1B). The initial detection of viral genomes in both serum and saliva typically occurred at, or before, the onset of symptoms (fever and/or rash—yellow boxes).

**Fig 2:**
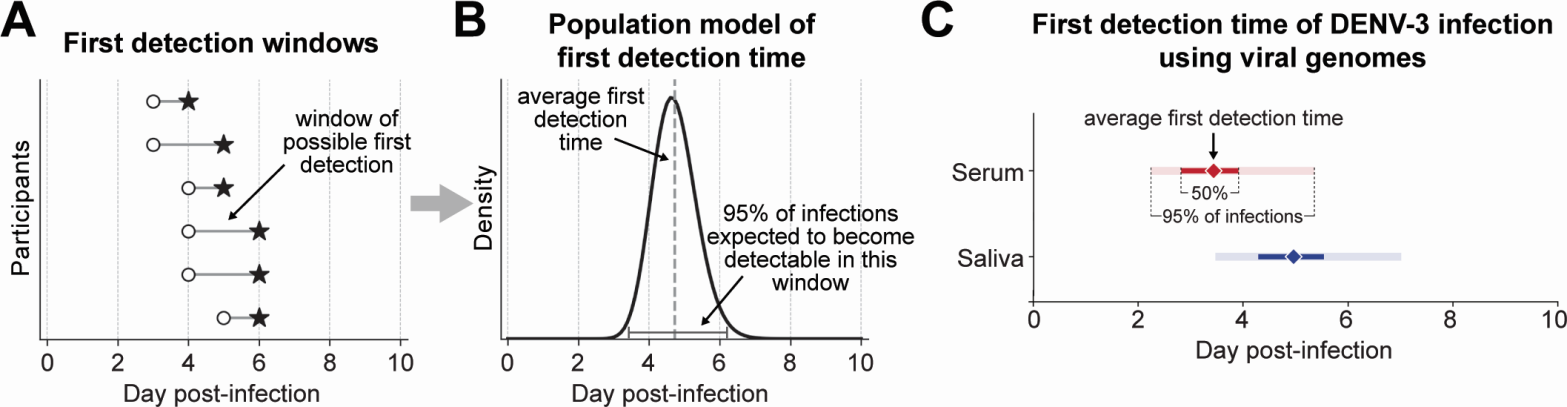
Timing of apparent DENV-3 genomes in blood versus saliva. (**A and B**) Schematics of a statistical model of the time until dengue virus genomes are first detectable by RT-qPCR. (**A**) For each participant, the first detectable time occurs within a window between the last day of non-detectable dengue genomes (open circle) and the first day of detectable dengue genomes (black star). Only six participants shown for illustrative purposes. (**B**) Maximum likelihood estimation of the parameters of the gamma distribution that best captures the observed data. (**C**) Summary statistics resulting from the statistical model of the first detectable time (all participants) using viral RNA from either serum or saliva.

The strengths of our study are the known date of infection and the subsequent repeated sampling scheme. To take advantage of these strengths, we estimated as a measure of sensitivity how soon after infection DENV-3 genomes could be detected in serum versus saliva. We built a statistical model that allowed us to address the limitation that samples were collected only once per day, yet the true first time (in hours post infection) that DENV-3 genomes become detectable falls somewhere between the last negative and the first positive specimen (Fig. 2A). We modeled the first detectable time as a gamma distribution, and then fit it to our data to find the shape, scale, and location parameters that are most consistent with our observations, using maximum likelihood estimation. Our estimates of first detectable times therefore incorporate the uncertainty and variability inherent in our data (Fig. 2B). This model allowed us to estimate that viral RNA is detectable in serum 1.6 days earlier on average than in saliva (Fig. 2C). Blood-based tests should be able to detect infection at 3.4 days post infection while saliva-based tests should be able to detect infection at 5.0 days post infection (Table S2). Confidence intervals for these estimates are given in Table S2.

### Host transcriptional responses to DENV-3 infection, as measured in both blood and saliva

**Fig 3:**
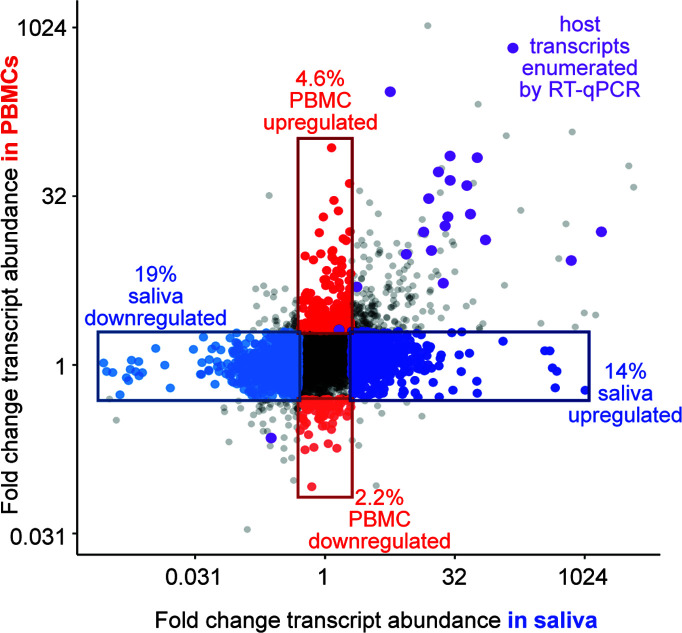
Host transcriptional responses to dengue virus infection. RNA sequencing analysis of PBMC and saliva samples from participants 1 and 5. For each human gene (dots), day 8 post-infection transcript levels were normalized to day 0 transcript levels. Transcripts that decreased or increased in abundance after infection only in PBMCs are boxed in red. Transcripts that decreased or increased only in saliva are boxed in blue. The percentage of human genes in each box is indicated. Dots in the upper right quadrant represent genes whose transcripts increased after infection in both biospecimens. Purple dots indicate host genes from which transcripts are quantified by RT-qPCR in further figures. Data shown are a composite of two biological replicates (two study participants), along with two technical replicates for each participant (each RNA sample was split in half and underwent two separate library preparations).

We also used our longitudinal study to acquire new knowledge about the host response to infection. We wished to identify human genes upregulated upon dengue infection. What is unique here is that, due to the nature of the challenge study, we have participants in which infection is synchronized with respect to timing, dose, and viral variant. To begin to utilize this resource, we first sequenced total RNA isolated from PBMC and saliva samples at days 0 and 8 for the biospecimens of two participants (1 and 5). For each participant, two technical replicates were performed for each participant (each RNA sample was split in half and underwent two separate library preparations). Technical replicates were collapsed by summing their counts prior to analysis. The resulting data for the two biological replicates (i.e.*,* participants) were analyzed using DESeq2 (29), which fits a negative binomial generalized linear model to the count data and calculates log_2_ fold change for each transcript from day 0 to day 8 post-infection, which was then plotted (Fig. 3; Fig. S1). DESeq2 borrows information across genes to stabilize variance estimates, which is particularly important when the number of biological replicates is small (29). Human genes from which transcripts increased or decreased in abundance after DENV-3 infection were defined by a twofold or greater increase or decrease on day 8 compared with day 0.

The upper right quadrant of the graph (Fig. 3) contains genes from which transcripts were increased in both PBMCs and saliva, representing 5.5% of all human genes. As expected, many of these genes are related to known host responses to viral infection (gene ontology analyses in Fig. S1). We also note that the other three quadrants on the graph are mostly empty. This is reassuring because two of these quadrants would contain genes representing an unexpected pattern: human genes detected as having increased transcript abundance in one biospecimen and decreased abundance in the other. Thus, we interpret this to mean that the noise in our data is low.

An unexpected signature that emerged is that many host genes were detected as having increased transcript abundance in only PBMCs (4.6%) or only saliva (14%) rather than in both biospecimens. These genes fall in the blue or red boxes along the axes in Fig. 3. Table 1 lists the top 30 human genes with increased transcript abundance by day 8 of dengue infection, as measured in PBMCs, saliva, or both. Many of these genes encode known restriction factors or regulators of the immune response (highlighted in bold).

**TABLE 1:** Top 30 human genes upregulated upon dengue infection

Increased expression in PBMC			Increased expression in saliva			Increased expression in both		
Human gene	Gene function	Day 8 fold change	Human gene	Gene function	Day 8 fold change	Human gene	Gene function	Day 8 fold change^*a*^
**CCL2**	**Chemokine^*d*^**	**87**	TRIB2	Signal transduction	1,000	NT5C3A	Nucleotide metabolism	1,900
ETV7	Transcription factor	42	LRRC8C	Anion channel	490	**SIGLEC1**	**Macrophage binding**	1,700
**C1QC**	**Complement system**	**29**	PI4K2A	Lipid synthesis	470	**IFI44^*b*^**	**Unassigned; interferon induced**	800
LAMP3	Autophagocytosis	24	CENPC	Centromere component	430	TFEC	Transcription factor	690
AMOTL2	Angiogenesis regulator	21	CWC22	Spliceosome component	400	ENSG00000289768	Unassigned	540
**APOBEC3A**	**Restriction factor**	**15**	GOLGA5	Golgi structure regulator	350	LYSMD2	Unassigned	510
TGM2	Apoptosis	15	DNAJC13	Endosome trafficking	120	OTOF	Calcium sensor	430
ABTB2	Unassigned	13	GPR27	Unassigned	58	IFI27^*b*^	Adapter protein	410
**APOBEC3B**	**Restriction factor**	**13**	ULK3	Autophagy regulator	58	**AZI2**	**Restriction factor**	400
**CXCR2**	**Chemokine receptor**	**11**	MAPK7	Transcriptional regulator	47	**RTP4^*b*^**	**Restriction factor**	360
HERC6	Ubiquitin ligase	10	**BTN3A3**	**T-cell response**	**39**	BATF2	Transcription factor	320
JUP	Structural	9.8	AMZ2	Unassigned	38	**DDX60**	**Restriction factor**	260
VSIG10L	Unassigned	9.4	RNF138	DNA repair	36	**TAFA2**	**Chemokine**	160
AGRN	Calcium receptor	9.1	PRKCZ	Signal transduction	30	**CXCL10^*b*^**	**Proinflammatory cytokine**	140
ADM	Angiogenesis regulator	8.7	**SP140**	**Unassigned; interferon induced**	**29**	**CCL8**	**Chemokine**	140
FOXC1	Transcription factor	8.6	CDK17	Unassigned	22	**IFIT1**	**Restriction factor**	120
**FCGR1A**	**IgG receptor**	**8.5**	ZMYND8	Transcriptional regulator	22	**CMPK2**	**Restriction factor**	110
MARCKS	Actin regulator	7.6	TARBP1	RNA binding	21	**RSAD2^*b*^**	**Restriction factor**	64
**FPR2**	**Chemokine receptor**	**7.0**	ZFX	Transcriptional regulator	21	**DHX58**	**Restriction factor**	63
**IL1RN**	**IL1 receptor agonist**	**6.9**	ASPRV1	Epidermis maintenance	18	**IFIT3^*b*^**	**Restriction factor**	51
**CX3CR1**	**Chemokine receptor**	**6.6**	TCEA1	Translation elongation	17	**IFI44L^*c*^**	**Restriction factor**	47
STAT1	Transcription factor	6.5	REEP3	Microtubule binding	17	**OAS2^*b*^**	**Restriction factor**	43
HELZ2	Transcription factor	6.1	M6PR	Lysosome function	16	**IFIT2^*b*^**	**Restriction factor**	42
TYMP	Angiogenesis regulator	5.8	NDUFA5	Oxidative metabolism	16	**USP18**	**ISG15 protease**	40
MYOF	Membrane repair	5.7	SLC3A2	Translocator	16	**SERPING1^*b*^**	**Complement system**	37
LRRC4	Adhesion	5.7	DNAJC7	Response to heat stress	15	**ISG15^*b*^**	**Immune response**	36
GCH1	GTP metabolism	5.4	AP3S1	Vesicle budding	15	**MX1^*b*^**	**Restriction factor**	35
SLC31A2	Copper ion import	5.2	EVL	Actin regulator	15	**CXCL9**	**Chemokine**	35
CEACAM3	Granulocyte receptor	5.1	GON4L	Transcriptional regulator	14	ARHGEF10L	Signal transduction	32
CDKN1A	Cell cycle regulator	4.9	CREB3L2	Transcription factor	14	TRMT1L	RNA binding	32

^*a*^ Average fold increase across both biospecimens.

^*b*^ These genes are included in the RT-qPCR panel described herein.

^*c*^ This gene, IFI44L (interferon induced protein 44 like), is related to another gene that has increased transcript abundance in both saliva and PBMCs, IFI44 (interferon induced protein 44), which is included in the RT-qPCR panel described herein.

^*d*^ Genes in bold text encode restriction factors or regulators of the immune response.

**Fig 4:**
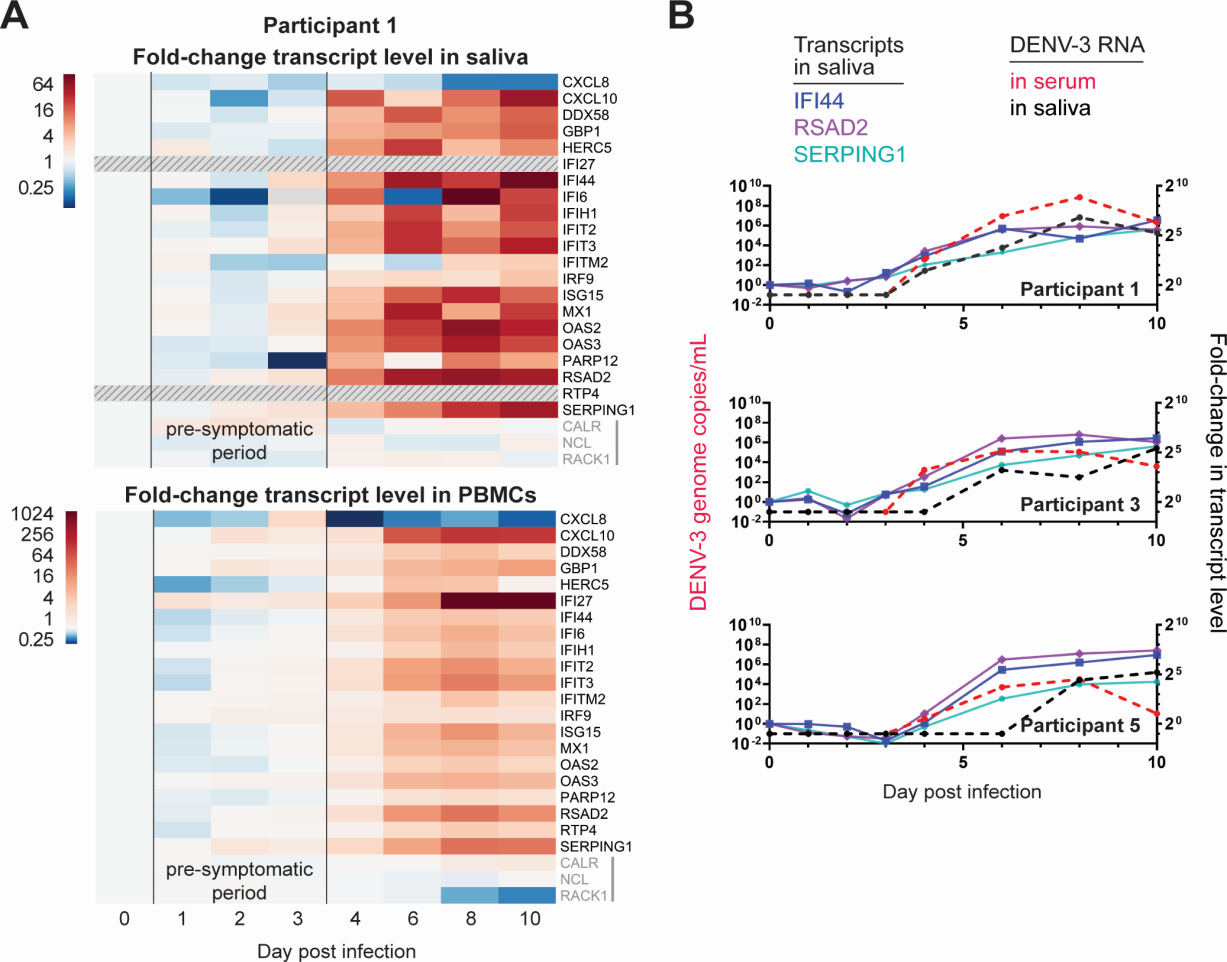
The kinetics of human gene upregulation in response to DENV-3 infection. (**A**) Total RNA was extracted from saliva and PBMCs from Participant 1, and RT-qPCR was used to quantify transcripts from 21 different human genes (identified in Fig. 3), and three housekeeping genes (right side, housekeeping genes in gray type). Similar data for all participants are shown in Fig. S2 and S3. Heatmaps show abundance of each mRNA transcript, after normalizing to the geometric mean of the Cq value of the housekeeping genes’ mRNA. Gray hashes indicate samples for which fold-change could not be calculated. The pre-symptomatic period was defined by days after infection each participant did not experience either fever or rash. (**B**) For three participants, the fold change in transcript abundance in saliva after infection is shown for three selected genes (*IFI44*, *RSAD2*, and *SERPING1*—solid lines), plotted alongside serum and saliva viral genome copies (red and black dashed lines, respectively).

By day 8 after infection, 5.5% of human genes had increased transcripts in both PBMCs and saliva (upper right quadrant of Fig. 3). We chose 21 of these to study further, with the goal of tracking the kinetics of upregulation of these genes across the course of DENV-3 infection in all study participants. Eleven of these 21 genes are in the “top 30” most highly upregulated genes in both PBMCs and saliva (Table 1), while the remaining 10 are upregulated in both samples but to a lesser degree. A total of eight multiplexed RT-qPCR reactions were performed on total RNA isolated from both saliva and PBMC from each participant, at each timepoint, with each done in technical duplicates. This resulted in 5,424 RT-qPCR reactions being performed. Transcripts from three housekeeping genes (*CALR*, *NCL*, and *RACK1*) were monitored in all samples as controls, and we previously established that these genes do not respond to infection (16). Except for the housekeeping genes, almost all the host genes were confirmed to increase over time in both saliva and PBMCs of all study participants (Fig. 4A; data from all participants shown in Fig. S2 and S3). For at least some of these host transcripts, the timing of detectable viral RNA in serum coincides with the increase of host transcripts in saliva (Fig. 4B), suggesting that the host response to infection is in communication across these two biological fluids. Interestingly, the increase of host transcripts in saliva sometimes occurred prior to detectable DENV-3 RNA in saliva.

## DISCUSSION

We used matched blood and saliva samples from nine participants of a DENV-3 human challenge study to show that DENV-3 genomes can be detected in saliva at similar titers and timepoints as in serum. Why dengue virus genomes are present in the saliva of infected individuals is not clear. We do not know if the dengue genomes detected here originate from infectious virions, non-infectious virions, or another source. One theory is that these dengue virus genomes are associated with cellular debris that is translocated to the oral cavity for elimination through the GI tract. Another possibility is that the genomes were from dengue virus-infected cells circulating in the bloodstream and introduced to the oral cavity through petechiae, a mild hemorrhagic symptom (30–33). Though bleeding or plasma leakage was not observed in any of the participants (22), we currently have no way of determining the true source of these genomes, and this would be an intriguing topic for future studies. Interestingly, a recent study also found detectable viral genomes of another blood-borne arbovirus, Crimean-Congo hemorrhagic fever virus (CCHFV), in the saliva of infected individuals (34). Also, previous studies have found Zika virus genomes as well as infectious Zika virus present in the saliva of symptomatic infected individuals (35–37).

We also used our longitudinal study to acquire new knowledge about the host response to infection. We wished to identify human genes upregulated upon dengue infection, and to determine during which time points after infection these changes take place. Transcripts from 5.5% of human genes increased in abundance by eight days of infection in both PBMCs and saliva, and many of these genes are known to be involved in the host immune response to infection (Table 1; Fig. S1). We chose to further enumerate a subset of these transcripts by RT-qPCR so that we could track their kinetics across the course of DENV-3 infection in all study participants (Fig. 4; Fig. S2 and S3). We generated a list of host genes whose transcript levels are increased upon infection and enumerated the kinetics with which transcripts accumulate in both saliva and in PBMCs. This adds to our previous studies on pathogen-specific host biomarkers that accumulate in saliva (16).

A striking signature that emerged is that many host genes were detected as having increased transcript abundance in only PBMCs or only saliva but not both. Given that dengue virus is a blood-circulating pathogen, it is perhaps easier to understand the host transcripts that have increased abundance specifically in PBMCs. The PBMC fraction contains monocytes and B-lymphocytes, both important targets of dengue virus infection, but not neutrophils (38, 39). Even more genes were seen to be upregulated in saliva compared with blood. The cellular composition of PBMC versus saliva is overlapping but distinct. Saliva contains a broader mix of cell types compared with PBMC, and this could influence our finding. Whole saliva contains a wide variety of cell types (40–43). Cells in the oral cavity could be responding to interferon, increasing the abundance of interferon-induced transcripts in those cells. Perhaps the complex cellular mix in the oral cavity provides a “wide net” and permissive signal amplifier for inflammatory processes taking place in the body. We note that the gene ontology categories of genes from which transcripts are enriched in PBMCs versus saliva are distinct (Table 1; Fig. S1). Future work could further elucidate the separate host responses to infection at play in each biospecimen.

There are several caveats to consider when interpreting the data from this human challenge study. First, the DENV-3 strain used in this study, CH53489, was developed as a challenge virus by serial passaging in monkey and mosquito cells (27). While this virus was found to cause dengue fever in human volunteers from multiple challenge studies, the timing and/or level of detectable viral genomes and induction of the host response could differ as compared to a wild-type dengue virus. Second, DENV-3 was delivered to the participants by needle (subcutaneous inoculation) rather than by an infected mosquito, which would more closely resemble natural infections. Previous research has shown that mosquito saliva carries important immunomodulatory factors that can alter or enhance the transmission of arboviruses, including dengue viruses (44–47), and these factors cannot be taken into consideration in the current study. Third, all participants in this study developed symptoms, whereas most natural dengue virus infections are asymptomatic (48, 49).

Blood-borne pathogens, such as dengue virus, bring with them a special barrier to medical diagnostics, surveillance, and epidemic control due to the requirement for a human blood draw. Our data suggest that saliva could be used as an alternative biological sample to detect dengue virus genomes in infected individuals, even early in the course of infection. Unlike blood, saliva is non-invasive, can be self-collected, does not require the use of sharps, and is thermostable (50–52). These traits make saliva an appealing and low-cost alternative to blood for detecting infection, and our study raises the possible utility of saliva in detecting dengue virus infection within 5 days post-infection.

## MATERIALS AND METHODS

### Saliva and blood samples collected from DENV-3 human challenge study

Nine participants were enrolled in this DENV-3 human challenge study (clinical trial NCT04298138) (22). These participants were determined to be naive to orthoflavivirus infection and healthy at the time of enrollment. Participants were subcutaneously inoculated with 0.5 ml of a 1.4 × 10^3^ PFU/mL suspension of DENV-3 strain CH53489. Blood and saliva samples were collected pre-infection for day 0, and subsequently on days 1, 2, 3, 4, 6, 8, and 10 post-infection. Not all participants provided blood and saliva samples on all days; Table S1 describes all samples available for this study.

Whole blood samples were collected using methods previously described (22, 23). From these whole blood samples, PBMCs were isolated by methods previously described (22) and stored in 90% FBS and 10% DMSO freezing media at −80°C. Serum isolation and viral genome quantification within were previously described (22).

We and others have previously shown that high-quality RNA can be recovered from saliva despite the nucleases and other challenges of this biospecimen (16, 43, 50, 53–55). The key is the device in which saliva is collected, Oragene CP-190 (DNA Genotek), which contains a stabilization buffer. This stabilizes the RNA at wide temperature ranges to preserve RNA integrity and is compatible with many traditional downstream applications (56). For each saliva sample, participants provided 2 mL of saliva, which was then mixed with 2 mL of stabilization buffer in the device. Samples were then incubated at 50°C for 1 h in a water bath per device manufacturer protocol and stored at −80°C.

### Extraction and RT-qPCR of DENV-3 viral RNA from saliva samples

RT-qPCR was used to detect and quantify dengue virus genomes in all saliva samples. DENV-3 RNA was extracted using the Zymo Research Quick-RNA Viral Kit (R1035). For each sample (Participant and Day), 400 μL of saliva and CP-190 buffer mixture was thawed and used for viral RNA extraction. As an extraction control, a monocytic cell line (U937-DC-SIGN cells; ATCC: CRL-3253) infected with dengue virus (DENV-2 Thailand/16,681/84) was suspended in 400 μL of CP-190 buffer and incubated at 50°C for 1 h before undergoing the same viral extraction protocol. The DENV-2 genomes from this extraction control were never quantified by RT-qPCR; this control simply allowed us to confirm extraction of viral RNA and its quality.

Viral RNA-containing eluate was quantified and checked for purity by Thermo Fisher Scientific NanoDrop One before being used for RT-qPCR amplification of the DENV-3 genome with Applied Biosystems TaqPath 1-Step RT-qPCR Master Mix, CG (A15300). Samples were loaded in technical duplicates. A master mix of an 8-point DENV-3 genome standard curve was created using ATCC Synthetic Dengue Virus Type 3 RNA (VR-3230SD, Lot #70054425) and loaded in technical triplicates alongside the samples. The sequences of the forward and reverse primers were 3′-GGACTGGACACACGCACCCA-5′ and 3′-CATGTCTCTACCTTCTCGACTTGTCT-5′, respectively. These sequences were slight modifications from previously described DENV-3 primers (57). The sequence for the probe was 3′- ACCTGGATGTCGGCTGAAGGAGCTTG-5′, as previously described (57). RT-qPCR results were analyzed using GraphPad Prism 10.1.2.

### Extraction of total RNA from PBMC samples for bulk RNA sequencing and RT-qPCR

Total RNA was extracted from PBMC samples using the Qiagen RNeasy Mini Kit (74106) with on-column DNase I digestion from the Qiagen RNase-free DNase Set (79256). As an extraction control, total RNA was also extracted from a monocytic cell line (U937-DC-SIGN cells; ATCC: CRL-3253) infected with dengue virus (DENV-2 Thailand/16681/84) using the same protocol. For this extraction control, the DENV-2 infection served to induce expression of host transcripts (in particular, the host transcripts used in the detailed RT-qPCR panel) since these transcripts are expected to have increased abundance only after infection. By infecting the cell line before harvesting total RNA, we ensure the transcripts of these host genes are increased such that we can robustly quantify them and confirm proper total RNA extraction. Eluted total RNA was quantified by Thermo Fisher Scientific NanoDrop One. Genomic DNA digestion was confirmed using an Agilent TapeStation 4150 and TapeStation RNA ScreenTape Analysis (5067-5576 and 5067-5577).

### High-throughput sequencing of total RNA from saliva and PBMC samples

Total RNA from day 0 and day 8 saliva samples from Participants 1 and 5 was extracted using the DNA Genotek protocol (PD-PR-021) for the extraction of RNA using the Qiagen RNeasy Micro Kit (74004). Total RNA from day 0 and day 8 PBMC samples from Participants 1 and 5 was already extracted using methods described above. PBMC and saliva RNA were then processed using the routine DNase treatment protocol from the Invitrogen TURBO DNA-*free* Kit (AM1907), followed immediately by the New England Biolabs Monarch Spin RNA Cleanup Kit protocol (T2040). Resulting DNA-free RNA from all PBMC and saliva samples was quantified and assessed for quality and purity using a Thermo Fisher Scientific NanoDrop One, Agilent TapeStation 4150 with TapeStation RNA ScreenTape Analysis (5067-5576 and 5067-5577), and Thermo Fisher Scientific Qubit 4 Fluorometer with Qubit RNA BR Assay Kit (Q10210).

DNA-free RNA was sent to Azenta Life Sciences for library preparation and next-generation sequencing. Each RNA sample was split into duplicates which underwent separate library preparations. Samples first underwent poly-A enrichment using the NEBNext Poly(A) mRNA Magnetic Isolation Module (E7490) then were additionally depleted for globin (PBMC samples) or bacterial ribosomal RNA (saliva samples) using the Qiagen QIAseq FastSelect Kits (334376 and 334386, respectively). Libraries were then prepared using the NEBNext Ultra II Directional RNA Library Prep Kit for Illumina (E7760L) using the unique dual indexes from NEBNext Multiplex Oligos for Illumina (E7600S). To aid in downstream normalization, Ambion ERCC RNA spike-in mix (Thermo Fisher Scientific 4456740) was added at a 1:1,000 ratio to all libraries. Libraries were sequenced in 150-bp paired-end format using NovaSeq 6000 (Illumina). Each replicate was sequenced to a depth of 30 million reads.

To identify transcripts with changes in abundance upon dengue virus infection, raw RNA sequencing reads were obtained in FASTQ file format and quality trimmed using BBDuk (BBTools v38.5) (58). Trimmed reads were then mapped to the human transcriptome from the hg38 assembly and quantified using Salmon (v1.10.3) (59). Transcript counts were then normalized based on ERCC spike-ins to account for differences in library size using the R (v4.4.0) package RUVSeq (v1.38.0). Samples were next separated based on biospecimen (PBMC or saliva) and the differential expression of genes by day 8 post-infection relative to day 0 was determined using the R package DESeq2 (v1.44.0) Wald test, using Participants 1 and 5 as biological replicates and the separate library preparations as technical replicates (29). Fold changes in transcript abundance were obtained from the DESeq2 results and plotted using ggplot2 (v3.5.1) (60). Gene ontology analyses of host transcripts were performed using the clusterProfiler package (v4.12.0). Gene functions in Table 1 were determined from NCBI and UniProtKD/Swiss-Prot gene summaries.

### Extraction of total RNA from saliva samples for RT-qPCR

Total RNA was extracted using a modified protocol based on the Zymo Research Quick-RNA Microprep Kit (R1051). As an extraction control, total RNA from pooled saliva of healthy donors was extracted alongside each participant’s samples. For each sample, 400 μL of saliva and CP-190 buffer mixture was thawed at room temperature then incubated at 75°C for 15 min. Samples were next put on ice for 5 min, then finally equilibrated to room temperature for 2 min. Then, 400 μL of RNA lysis buffer was added to the 400 μL sample mixture, which was then processed using the Zymo Research Quick-RNA Microprep protocol.

Eluted total RNA was quantified and checked for purity using a Thermo Fisher Scientific NanoDrop One. Additionally, we checked for mRNA degradation and genomic DNA contamination using an Agilent TapeStation 4150 and TapeStation RNA ScreenTape Analysis (5067-5576 and 5067-5577).

Despite multiple extraction attempts, Participant 2’s saliva produced viral RNA of marginal quality and total RNA of poor quality. Confounding variables such as improper volumes of saliva provided upon collection, eating or drinking within 30 min before collection, or individual variability could explain Participant 2’s less than ideal saliva RNA. Therefore, we included Participant 2 in our viral RNA analyses but excluded them from any host mRNA analyses.

### RT-qPCR for enumerating human transcripts

A total of eight multiplexed RT-qPCR reactions were performed for each sample (RNA from saliva or PBMC from a given individual at a given timepoint) in technical duplicates. Each multiplexed reaction evaluated three target genes, for a total of 24 host transcripts analyzed for each sample. Three of these transcripts were used as housekeeping controls (*CALR*, *NCL*, and *RACK1*).

The multiplexed RT-qPCR reactions were prepared with Applied Biosystems TaqPath 1-Step RT-qPCR Master Mix, CG (A15300) and custom primer/probe mixes using ROX, VIC, FAM, and ABY fluorescent channels. Samples were run on the Thermo Fisher QuantStudio 3 qPCR machine.

Raw RT-qPCR Cq values were imported into R (v4.4.0) for analysis. First, Cq values of technical replicates were averaged. For each sample, the Cq values of the three housekeeping controls were averaged to reduce the effects of noise in the housekeeping transcripts’ abundance (61, 62). We then calculated the ΔCq for all 21 host genes (targets) on each day of the study as ${∆Cq}_{target}={Cq}_{target}-{Cq}_{hkg}$, where Cq_target_ represents the averaged Cq value for a particular host transcript and Cq_hkg_ represents the averaged Cq for all three housekeeping transcripts. Finally, we calculated the log_2_ fold change in transcript abundance for all host genes relative to their expression on day 0, when the participants were healthy. To find the log_2_ fold change (log_2_FC) of a transcript abundance on day M post-infection, we used the equation ${log}_{2}FC(target,DayM)={∆Cq}_{targetDay0}-{∆Cq}_{targetDayM}$. From this result, fold changes in host transcript abundance were calculated and generated into heatmaps using the pheatmap package (v1.0.12) in R (v4.4.0) and Adobe Illustrator 28.3.

### Statistically modeling time of first detection

For each participant, the time at which the dengue genome first becomes detectable by RT-qPCR lies between the time of the first positive sample and the sample that preceded it. On this premise, we developed a statistical model to infer the distribution of first detectable times at the population level from the observed data. We defined *t_d_* to be the first time at which an individual becomes detectable for DENV-3 infection given detection of virus genome. These estimates of *t_d_* allowed us to compare first detectable times between different biological samples.

For each participant’s measurements of viral RNA (in serum or saliva), we recorded the times of the last negative ($t^{-}$) and first positive ($t^{+}$) samples, where a positive sample is one in which viral RNA concentration exceeds the detection threshold. Viral genomes were quantified in serum and saliva samples using a highly sensitive RT-qPCR assay with a limit of detection around 10 copies/mL. Thus, the detection threshold for viral RNA was set to 10 copies/mL. By definition, the precise time of first possible detectability, *t_d_*, occurred sometime between the observed $t^{-}$ and $t^{+}$. We assumed that *t_d_* follows a gamma distribution at the population level, with shape, scale, and location parameters to be informed by our data. Under these assumptions, the likelihood L of observing the participants’ $t^{-}$ and $t^{+}$ is given by the following:


$$L=\prod_{i=1}^{n}\left[\int_{{t_{i}}^{-}}^{{t_{i}}^{+}}f\left(t|k,\theta ,\alpha \right)dt\right],$$

where f(t | k,θ,α) is the gamma distribution probability density function parameterized using the shape parameter k, the scale parameter θ, and the location parameter α. We used the scipy optimize dual annealing routine (v1.14.1) in Python (v3.9.6) to estimate the values of the model parameters (k,θ,α), which maximize the likelihood function given the observed data.

Our analysis relied on n=9 samples for viral genome data. For individuals whose samples never crossed the threshold for detectability, $t^{+}$ was considered to be infinite, such as Participant 9 who provided saliva only up to day 4 post-infection.

All calculated population distributions for first detectable time are shown in Table S2.

## Data Availability

High-throughput sequencing data (RNA-seq) are available under GEO accession GSE324729.

## SUPPLEMENTAL MATERIAL

Supplemental material jvi.00373-26-s0001.pdf Tables S1 and S2; Fig. S1 to S4.

ASM does not own the copyrights to Supplemental Material that may be linked to, or accessed through, an article. The authors have granted ASM a non-exclusive, world-wide license to publish the Supplemental Material files. Please contact the corresponding author directly for reuse.
