# Supplementary material for: Longitudinal analysis of viral and host RNA in blood and saliva during controlled human dengue virus 3 infections
Source: J Virol. 2026 May 14;100(6):e00373-26. doi: 10.1128/jvi.00373-26 (PMC13289089; doi:10.1128/jvi.00373-26)
Supplement: Supplemental material — Tables S1 and S2; Fig. S1 to S4. [file jvi.00373-26-s0001.pdf]

Longitudinal analysis of viral and host RNA in blood and saliva during controlled human dengue virus 3 infections

SUPPLEMENTAL INFORMATION

Sharon S. Wu[a,b], Jennifer N. Bjerke[c], Casey E. Middleton[b,d], Arturo Barbachano-Guerrero[a,b], Joshua R. Dye[c], Emma R. Worden-Sapper[a,b], Amy B. Emerman[c], Timothy P. Endy[e], Stephen J. Thomas[e,f], Adam T. Waickman[e,f], Daniel B. Larremore[b,d,g], Nicholas R. Meyerson[c], Sara L. Sawyer[a,b]#

		Day post-infection										
		**0**	**1**	**2**	**3**	**4**	**5**	**6**	**7**	**8**	**9**	**10**
**Participant 1**	Serum viral RNA	x	x	x	x	x		x		x		x
	Saliva viral RNA	x	x	x	x	x		x		x		x
	PBMC total RNA	x	x	x	x	x		x		x		x
	Saliva total RNA	x	x	x	x	x		x		x		x
**Participant 2**	Serum viral RNA	x	x	x	x	x		x		x		x
	Saliva viral RNA	x	x	x	x	x		x		x		x
	PBMC total RNA	x	x	x	x	x		x		x		x
	Saliva total RNA	x	x	x	x	x		x		x		x
**Participant 3**	Serum viral RNA	x	x	x	x	x		x		x		x
	Saliva viral RNA	x	x	x	x	x		x		x		x
	PBMC total RNA	x	x	x	x	x		x		x		x
	Saliva total RNA	x	x	x	x	x		x		x		x
**Participant 4**	Serum viral RNA	x	x	x	x	x		x	x	x	x	x
	Saliva viral RNA	x	x	x	x	x		x				
	PBMC total RNA	x	x	x	x	x		x				
	Saliva total RNA	x	x	x	x	x		x				
**Participant 5**	Serum viral RNA	x	x	x	x	x		x		x		x
	Saliva viral RNA	x	x	x	x	x		x		x		x
	PBMC total RNA	x	x	x	x	x		x		x		x
	Saliva total RNA	x	x	x	x	x		x		x		x
**Participant 6**	Serum viral RNA	x	x	x	x	x		x	x	x		
	Saliva viral RNA	x	x	x	x	x		x				
	PBMC total RNA	x	x	x	x	x		x				
	Saliva total RNA	x	x	x	x	x		x				
**Participant 7**	Serum viral RNA	x	x	x	x	x		x		x	x	
	Saliva viral RNA	x	x	x	x	x		x				
	PBMC total RNA	x	x	x	x	x		x				
	Saliva total RNA	x	x	x	x	x		x				
**Participant 8**	Serum viral RNA	x	x	x	x	x		x		x	x	
	Saliva viral RNA	x	x	x	x	x		x				
	PBMC total RNA	x	x	x	x	x		x				
	Saliva total RNA	x	x	x	x	x		x				
**Participant 9**	Serum viral RNA	x	x	x	x	x		x	x		x	
	Saliva viral RNA	x	x	x	x	x						
	PBMC total RNA	x	x	x	x	x						
	Saliva total RNA	x	x	x	x	x						

Table S1. Samples available for participants in the DENV-3 challenge study. Blood and saliva were collected from each participant and processed, resulting in four types of samples used in this study: DENV-3 viral RNA in serum (serum viral RNA), DENV-3 viral RNA in saliva (saliva viral RNA), total RNA from PBMCs (PBMC total RNA), and total RNA from saliva (saliva total RNA). Sample boxes with an 'x' indicate that the sample was available and used in this study. Empty sample boxes indicate samples that were not collected or available for use. All serum viral RNA samples were quantified by Waickman et. al. (1). Note that while Participant 2 had saliva total RNA samples (gray boxes), we did not include them in this paper as they failed all quality controls even with multiple attempts at total RNA extraction. Additionally, we were unable to get saliva viral RNA or saliva total RNA data from Participant 7 Day 4 (blue boxes) as there was not enough saliva for viral RNA or total RNA extraction.

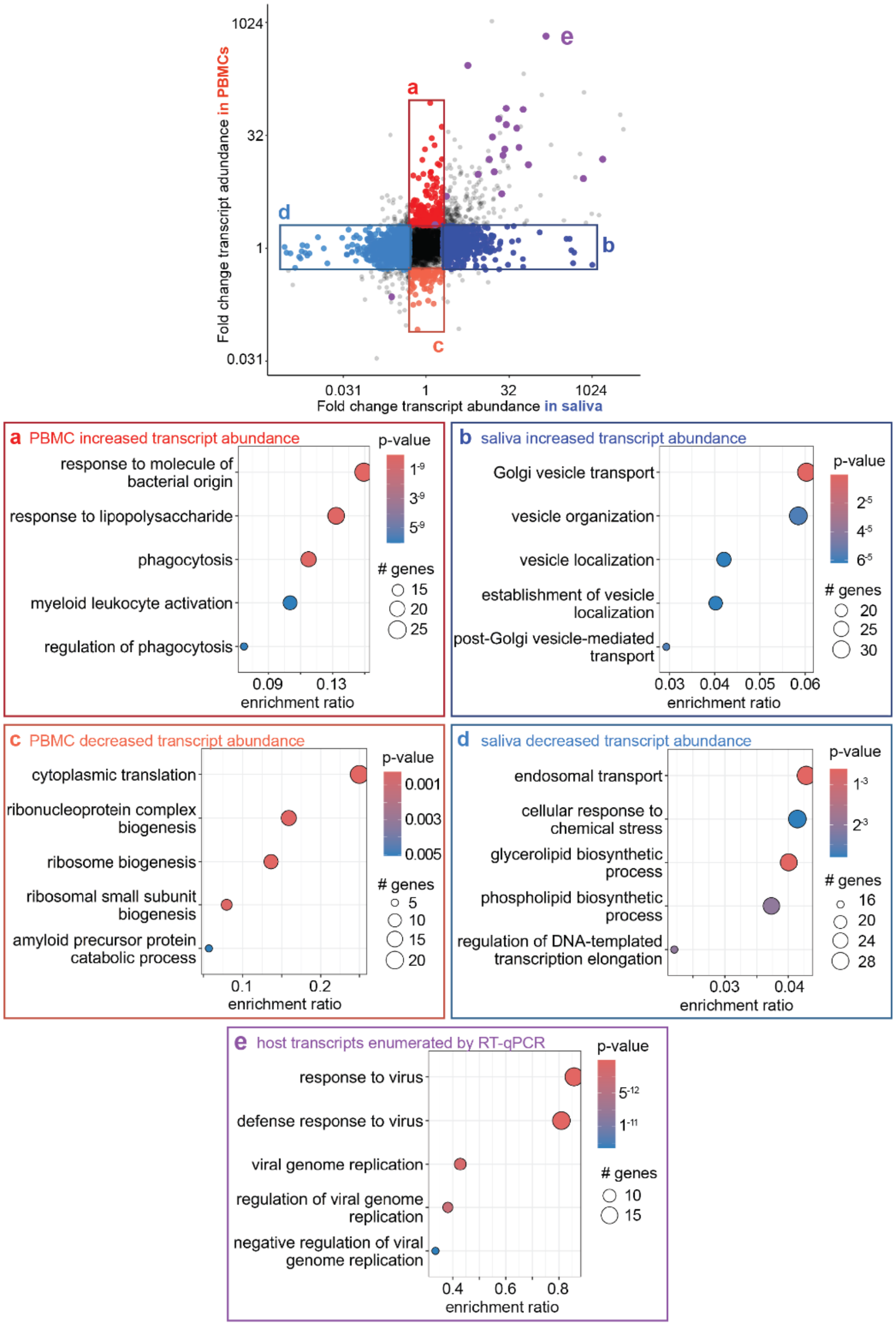

Figure S1. GO category analysis of host genes upregulated by dengue virus infection. Transcripts from day 8 post-infection PBMC and saliva samples from Participants 1 and 5 were normalized to their matched day 0 baseline levels. Fold change in transcript abundance in saliva is plotted along the X-axis, while fold change in transcript abundance in PBMCs is plotted along the Y-axis. Most transcripts can be grouped into one of four subsets: (a) increased abundance only in PBMCs, (b) increased abundance only in saliva, (c) decreased abundance only in PBMCs, or (d) decreased abundance only in saliva. However, the 21 host transcripts studied by RT-qPCR here (e) represent a distinct subset of transcripts which show similarly increased abundance in both PBMCs and saliva. The corresponding gene ontology (GO) annotations for each of these subsets is shown in the labelled panels. The enrichment ratio is calculated as the number of transcripts within the subset labelled with a GO category divided by the number of total transcripts associated with that GO category (*i.e.* the percent of transcripts in the GO category represented by the transcripts in the subset). P-values are calculated as the probability of observing the given number of transcripts in each GO category by random chance.

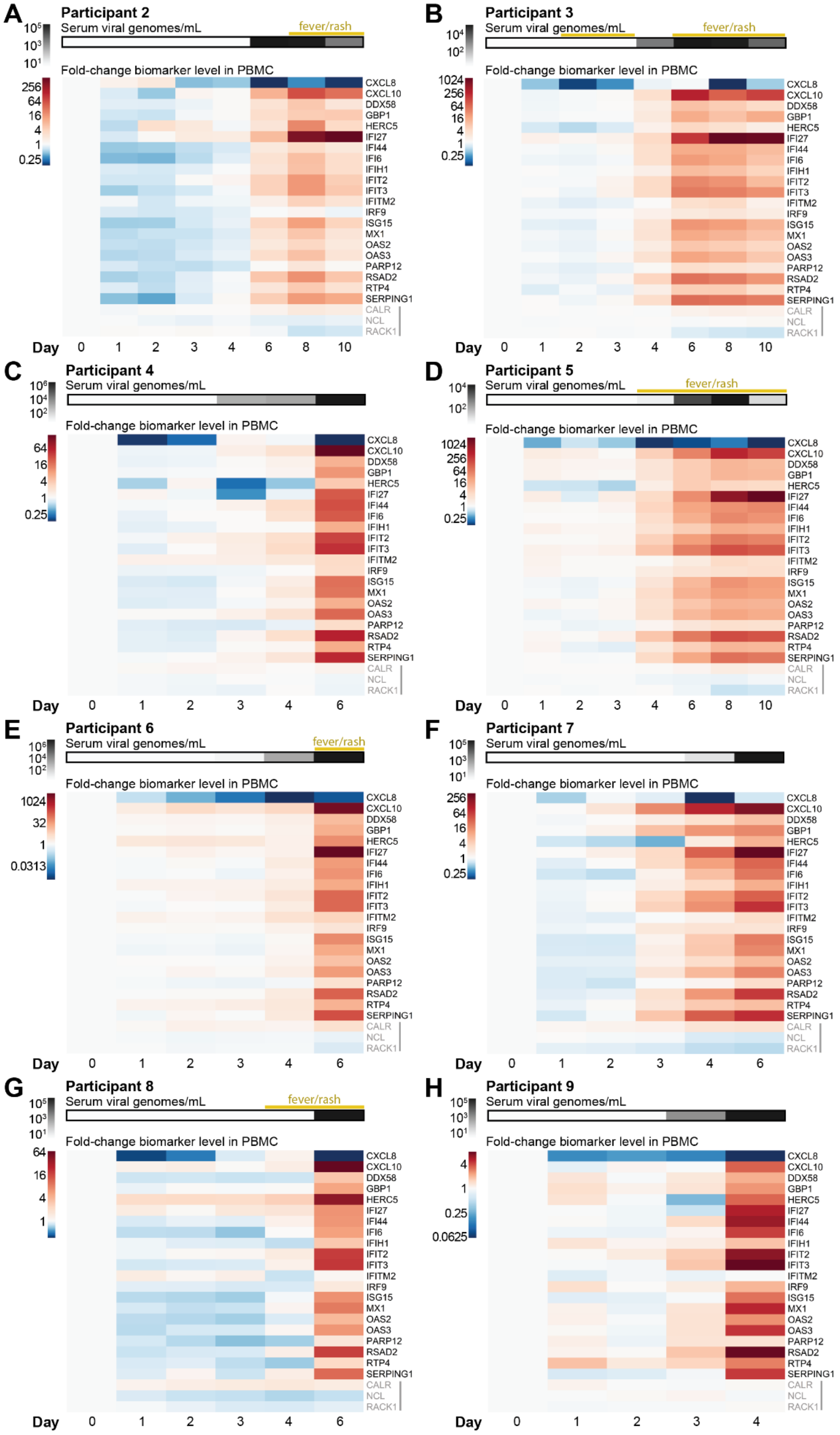

Figure S2. Transcriptional responses to dengue virus infection as measurable in blood. Heatmaps show host transcript abundance in PBMCs. Red and blue heatmaps show fold change in abundance for the 21 host transcripts (black type) and three housekeeping controls (gray type) from total RNA extracted from PBMCs. Above the heatmaps, the genome copies per milliliter (viral genomes/mL) as detected in serum (grayscale) is annotated. Days when participants experienced fever and/or rash are indicated with the yellow line.

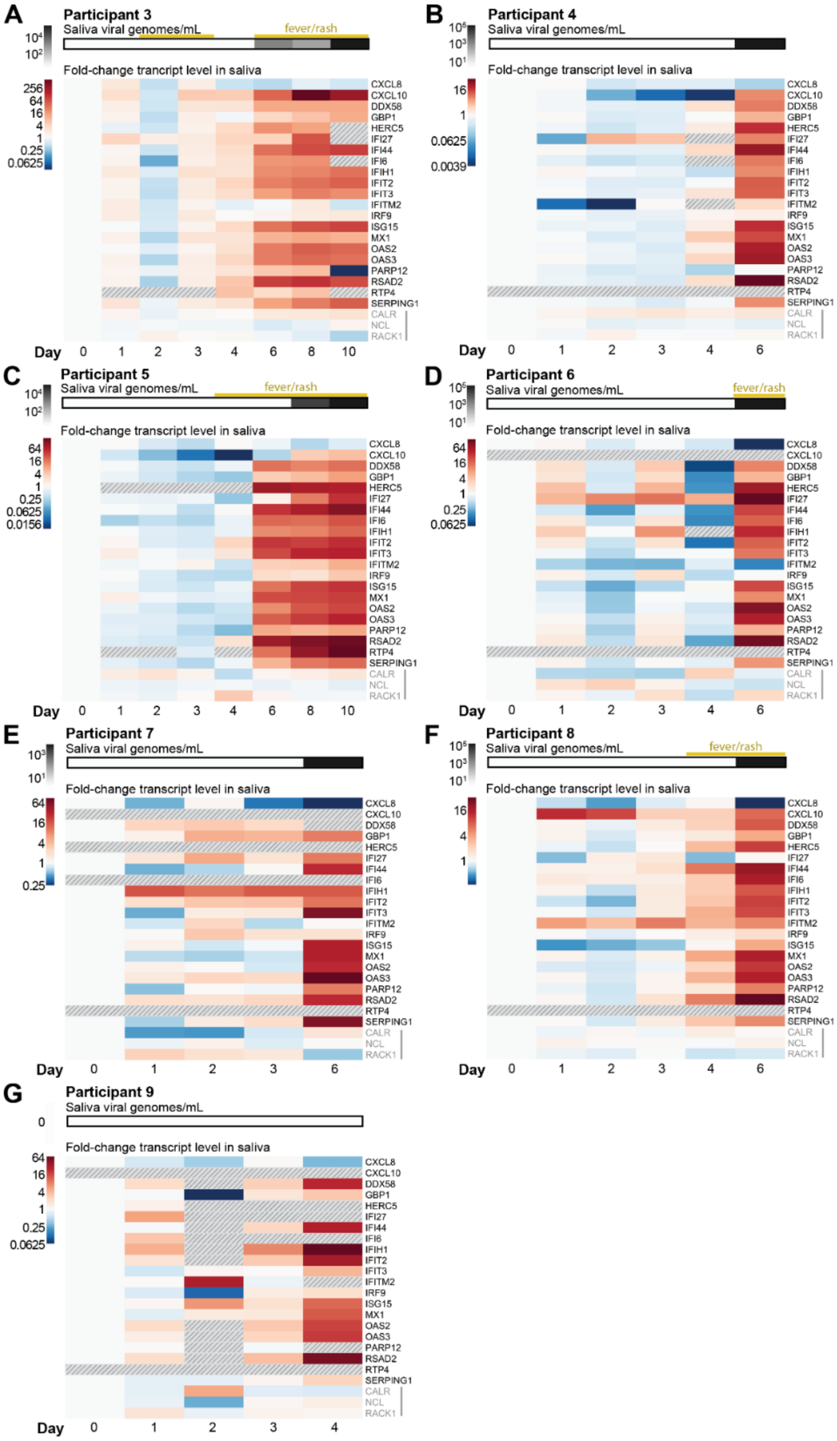

Figure S3. Transcriptional responses to dengue virus infection as measurable in saliva. Heatmaps show host transcripts from each gene (right), and DENV-3 genome copy, as measured in saliva. Red and blue heatmaps show fold change in abundance for the 21 host transcripts (black type) and three housekeeping controls (gray type) from total RNA extracted from saliva. Data for which fold change in transcript abundance was unable to be calculated is represented by boxes with gray hashes. Above the heatmaps, the genome copies per milliliter (viral genomes/mL) as detected in saliva (grayscale) is annotated. Days when participants experienced fever and/or rash are indicated with the yellow line. Participant 2 was not included in this analysis because the extracted total RNA from this individual's saliva did not pass multiple quality control checks.

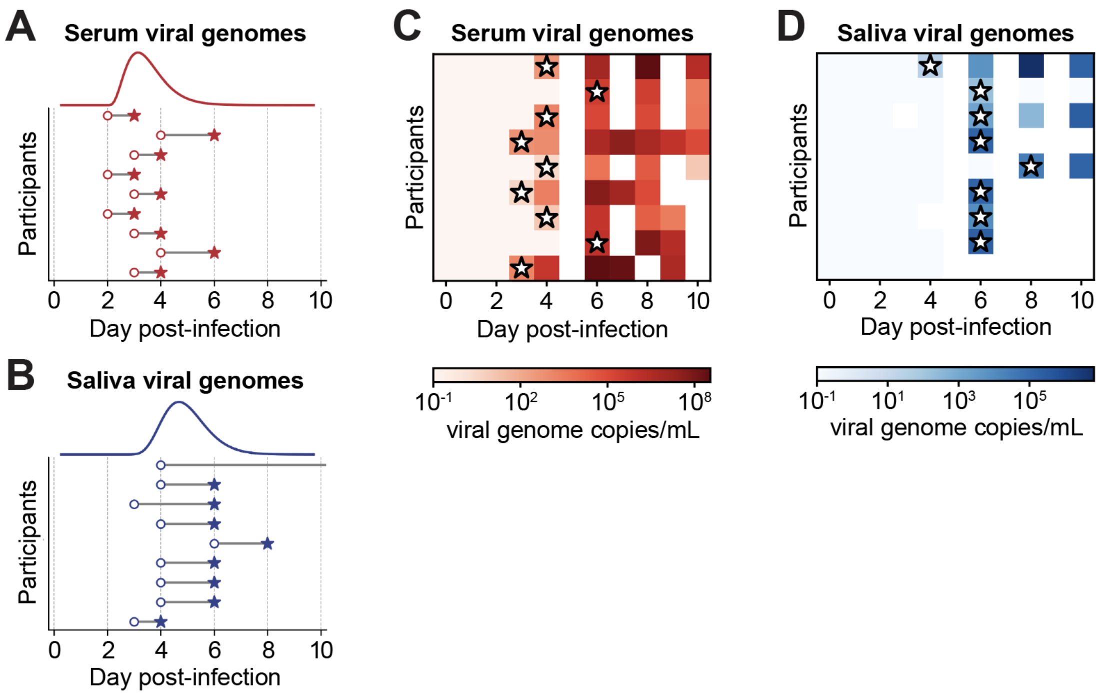

Figure S4. The day that DENV-3 genomes are first detected varies by participant and biospecimen. A,B) Windows of first detectable time between the last observed negative (ring) and first observed positive (star) tests were extracted from viral RNA for each participant and biological sample. Maximum likelihood estimation was used to estimate the parameters of the gamma distribution which best captures the observed data. Best-fit distributions are shown as curves atop observed last negative and first positive sample days using serum viral RNA measurements (A) and saliva viral RNA measurements (B). C,D) Time-varying quantification of viral genomes in serum and saliva (C and D, respectively). Darker colors represent higher quantification. White squares represent days with no sample quantification. Each row denotes a participant, in numerical order from top to bottom. The white stars represent the first detection time for DENV-3 infection by viral genomes for each participant.

RNA Source	Sample Type	Mean	Lower 95%	Upper 95%	Lower 50%	Upper 50%
Viral RNA	Serum	3.43	2.31	5.28	2.86	3.86
	Saliva	4.96	3.54	6.93	4.33	5.48

Table S2. Population distributions for first detectable times of DENV-3 genomes. Mean values were used for comparisons discussed within the body of the paper. Lower 95% and Upper 95% columns represent the lower and upper bounds of the equal-tailed interval containing 95% of each distribution, respectively, i.e. the 2.5th and 97.5th percentiles of first detectable times. Lower 50% and Upper 50% columns represent the lower and upper bounds of the equal-tailed interval containing 50% of each distribution, respectively, i.e. the 25th and 75th percentiles of first detectable times.

References

1. Waickman AT, Newell K, Lu JQ, Fang H, Waldran M, Gebo C, Currier JR, Friberg H, Jarman RG, Klick MD, Ware LA, Endy TP, Thomas SJ. 2024. Low-dose dengue virus 3 human challenge model: a phase 1 open-label study. Nat Microbiol 1–12.
